# HIF-1α/Beclin1-Mediated Autophagy Is Involved in Neuroprotection Induced by Hypoxic Preconditioning

**DOI:** 10.1007/s12031-018-1162-7

**Published:** 2018-09-10

**Authors:** Na Lu, Xingxing Li, Ruolan Tan, Jing An, Zhenlu Cai, Xiaoxuan Hu, Feidi Wang, Haoruo Wang, Chengbiao Lu, Haixia Lu

**Affiliations:** 1 Institute of Neurobiology, School of Basic Medical Sciences, Xi’an Jiaotong University Health Science Center, Xi’an, 710061 Shaanxi People’s Republic of China; 2 Key Laboratory for the Brain Research of Henan Province, Department of Physiology and Neurobiology, Xinxiang Medical University, Xinxiang, 453003 Henan People’s Republic of China

**Keywords:** Hypoxic preconditioning, Oxygen-glucose deprivation/reperfusion, Autophagy, HIF-1α, Beclin1, SH-SY5Y cells

## Abstract

Hypoxic preconditioning (HPC) exerts a protective effect against hypoxic/ischemic brain injury, and one mechanism explaining this effect may involve the upregulation of hypoxia-inducible factor-1 (HIF-1). Autophagy, an endogenous protective mechanism against hypoxic/ischemic injury, is correlated with the activation of the HIF-1α/Beclin1 signaling pathway. Based on previous studies, we hypothesize that the protective role of HPC may involve autophagy occurring via activation of the HIF-1α/Beclin1 signaling pathway. To test this hypothesis, we evaluated the effects of HPC on oxygen-glucose deprivation/reperfusion (OGD/R)-induced apoptosis and autophagy in SH-SY5Y cells. HPC significantly attenuated OGD/R-induced apoptosis, and this effect was suppressed by the autophagy inhibitor 3-methyladenine and mimicked by the autophagy agonist rapamycin. In control SH-SY5Y cells, HPC upregulated the expression of HIF-1α and downstream molecules such as BNIP3 and Beclin1. Additionally, HPC increased the LC3-II/LC3-I ratio and decreased p62 levels. The increase in the LC3-II/LC3-I ratio was inhibited by the HIF-1α inhibitor YC-1 or by Beclin1-short hairpin RNA (shRNA). In OGD/R-treated SH-SY5Y cells, HPC also upregulated the expression levels of HIF-1α, BNIP3, and Beclin1, as well as the LC3-II/LC3-I ratio. Furthermore, YC-1 or Beclin1-shRNA attenuated the HPC-mediated cell viability in OGD/R-treated cells. Taken together, our results demonstrate that HPC protects SH-SY5Y cells against OGD/R via HIF-1α/Beclin1-regulated autophagy.

## Introduction

Cerebrovascular diseases, such as stroke, have a high incidence of morbidity and mortality. Despite decades of extensive research and clinical practice, effective treatments for stroke are still limited; thus, current research continues to explore effective means of prevention (Jain et al. 2016). One possible therapeutic approach is the use of hypoxic preconditioning (HPC). HPC, also called hypoxia-induced tolerance, is defined as a phenomenon in which a brief period of hypoxia protects against subsequent, otherwise lethal insults (Stetler et al. 2014). HPC activates multiple endogenous protective mechanisms against severe or even fatal ischemic stroke and other acute attacks (Elena and Mikhail 2015). In vivo and in vitro studies have confirmed the protective effects of HPC against cerebrovascular stroke, but the mechanism behind this protection remains unclear (Lu et al. 2005; Yu et al. 2013). Studies have shown that HPC protection is eliminated in hypoxia-inducible factor-1α (HIF-1α) knockout mice subjected to neonatal hypoxia/ischemia (Sheldon et al. 2014). HPC induces tolerance to oxygen-glucose deprivation (OGD) in astrocytes, at least in part, via HIF-1α-linked upregulation of the P450 epoxygenase pathway (Liu and Alkayed 2005). HIF-1α is critically involved in ischemic preconditioning against stroke by modulating inflammatory responses in rats (Yang et al. 2018), suggesting that HIF-1α plays a key role in the regulation of HPC protection.

Autophagy is a lysosome-dependent degradation pathway that can clear away damaged organelles such as mitochondria and biological macromolecules including proteins, thus maintaining intracellular homeostasis (Denton et al. 2015). Rapamycin (Rapa) regulates autophagy through inhibition of the nutrient-sensing molecule known as mammalian target of rapamycin (mTOR) (Nair and Ren 2012). Phosphatidylinositol 3-kinase (PI3K) is a key regulator of autophagy, as it plays an important role in many biological processes, including controlling the activation of mTOR. 3-Methyladenine (3-MA) inhibits autophagy by blocking autophagosome formation via the inhibition of class III PI3K (Abdulrahman et al. 2011; Jin et al. 2015). The autophagy level is low under physiological conditions but can be increased under hypoxic conditions (Park et al. 2018); this increase can aggravate injury but can also play a protective role (Denton et al. 2015). The effective control of autophagy is important for the development of new treatment strategies for hypoxia-induced brain injury. Recent studies show that HPC plays a neuroprotective role by activating modest autophagy (Sheng et al. 2014; Wang et al. 2013b; Wang et al. 2014), but the mechanism involved is unclear.

The HIF-1α signaling pathway is closely related to hypoxia-induced autophagy (Wu et al. 2015). HIF-1 is a critical nuclear transcription factor in hypoxia adaptation and is composed of a labile HIF-1α subunit and a stable HIF-1β subunit (Loboda et al. 2012; Semenza 2012). HIF-1α is degraded by proteasomes under normoxic conditions. In response to hypoxia, however, HIF-1α is transferred into the nucleus and interacts with the HIF-1β subunit to form HIF-1 to initiate or enhance transcription of effector genes, including BNIP3/BNIP3L, insulin-like growth factor II, and vascular endothelial growth factor (Chu and Jones 2016; Xiong et al. 2015).

B cell leukemia/lymphoma 2 (Bcl-2)/adenovirus (E1B)-19KD-interacting protein 3 (BNIP3) is one of the Bcl-2 homology 3 (BH-3)-only proteins. BNIP3 is known to be involved in the regulation of both apoptosis and autophagy (Vasagiri and Kutala 2014). This protein activates autophagy by inhibiting the function of mTOR (Shi et al. 2014) and dissociates Beclin1 from Beclin1/Bcl-2 or Beclin1/Bcl-XL complexes by competing with Beclin1 during the formation of the autophagosome (Ney 2015). Accumulated evidence shows that the activation of autophagy is regulated by the HIF-1α/BNIP3 pathway (Lu et al. 2016; Wu et al. 2015; Zhao et al. 2012). Beclin1 is a well-known key regulator of autophagy; this protein controls the autophagic process by regulating class III PI3K-dependent generation of phosphatidylinositol 3-phosphate (PI3P) and the subsequent recruitment of additional autophagy-associated (ATG) proteins that promote autophagosome formation (Wirawan et al. 2012), which plays an essential role in autophagy activation (Maejima et al. 2016; Sinha and Levine 2008). Recent studies have confirmed that mimicking hypoxia with CoCl_2_ induces autophagy via activation of the HIF-1/BNIP3/Beclin1 pathway in skeletal myotubes (Chen et al. 2017b). In this study, we adapted an oxygen-glucose deprivation/reperfusion (OGD/R) model in SH-SY5Y cells and determined whether the HIF-1α/Beclin1 signaling pathway modulates autophagy and whether such regulation contributes to HPC-induced protection against OGD/R.

## Materials and Methods

### Cell Culture

Human SH-SY5Y neuroblastoma cells were purchased from the cell bank of the Chinese Academy of Sciences (Shanghai, China) and maintained in Invitrogen Dulbecco’s modified Eagle’s medium (DMEM; Thermo Fisher Scientific, Waltham, MA, USA) supplemented with 10% fetal bovine serum, 3.7 g/L sodium bicarbonate, 100 units/mL penicillin, and 100 g/mL streptomycin (Gibco-Thermo Fisher Scientific) at 37 °C in a tissue culture incubator with 5% CO_2_ and 95% relative humidity. We added 5 mM 3-MA (Sigma, St. Louis, MO, USA) or 25 mM Rapa (Cell Signaling Technology, Danvers, MA, USA) to inhibit or induce autophagy, respectively.

### OGD/R and HPC

Ischemic injury was induced in vitro using OGD/R, as described previously (Darshit and Ramanathan 2016; Guo et al. 2014; Zhu et al. 2016). Briefly, 5 × 10^4^/mL cells were maintained in glucose-free and serum-free DMEM in a hypoxia incubation chamber (Thermo, Forma 3427, USA) under hypoxic conditions (1% O_2_, 5% CO_2_, and 94% N_2_) for 10 h and then cultured under normoxic conditions (5% CO_2_, 95% air) in normal DMEM for a further 12 h. For HPC (Peterson et al. 2011; Tzeng et al. 2010; Wang et al. 2013b), cells were successively incubated (5% O_2_, 5% CO_2_, and 90% N_2_) for 9 h and under normoxic conditions for 12 h in normal DMEM. The control culture was always maintained in normal DMEM and put in the incubator under normoxic conditions.

### Cell Viability Assay

Cell viability was determined using MTT (3-(4,5-dimethyl-2-thiazolyl)-2,5-diphenyl-2H-tetrazolium bromide) assays. Briefly, 2 × 10^4^ cells per well were seeded in 96-well plates and incubated at 37 °C for 24 h. After the indicated treatment, 10 μL MTT (5 mg/mL; Sigma-Aldrich) was added to each well, followed by incubation for 4 h at 37 °C. Thereafter, the culture medium was replaced with 150 μL dimethyl sulfoxide (DMSO; Sigma-Aldrich) to dissolve any blue formazan crystals. After 10 min of incubation at room temperature, the optical density (OD) was measured at a wavelength of 570 nm using a microplate reader (BioTek Instruments, Inc., Winooski, VT, USA).

### Treatment with YC-1

3-(5′-Hydroxymethyl-2′-furyl)-1-benzylindazole (YC-1) (Cayman Chemical Company, Ann Arbor, MI, USA) was dissolved in DMEM containing 1% DMSO (Bu et al. 2011; Tsai et al. 2013; Yan et al. 2011). SH-SY5Y cells were pretreated with 10 μM YC-1 for 1 h before HPC (Tsui et al. 2013).

### In Situ One-step TUNEL

Cell apoptosis was determined using a terminal deoxynucleotidyl transferase-mediated dUTP nick end labeling (TUNEL) kit according to the manufacturer’s instructions (Nanjing KGI Biological Technology Development Co. Ltd., China). Briefly, cells were fixed with 4% paraformaldehyde in PBS for 1 h followed by permeabilization with 0.1% Triton X-100 in PBS for 10 min and then incubated with TUNEL detection solution at 37 °C for 1 h. Cell nuclei were counterstained with DAPI (Roche Molecular Systems, Inc., Pleasanton, CA, USA) for 20 min. TUNEL-positive cells had pyknotic nuclei with dark green fluorescent staining, indicative of apoptosis. Images were captured using a fluorescence microscope (Olympus IX71, Japan). Five random visual fields from each of five slides were observed at a magnification of × 200. TUNEL-positive cells were counted using Image-Pro Plus 5.0 (Media Cybernetics Inc., Rockville, MD, USA). The experiments were independently repeated three times.

### Flow Cytometric Evaluation of Apoptosis

Quantification of apoptotic cells was also performed by flow cytometry using Annexin V-fluorescein isothiocyanate (FITC) and propidium iodide (PI). In brief, cells were seeded in 24-well plates at a density of 1 × 10^5^ cells per well. Cells were harvested by trypsinization and gently washed twice with PBS after the various treatments, as indicated in the figures. The cells were stained with Annexin V-FITC using an Apoptosis Detection Kit (Beijing Biosea Biotechnology Co. Ltd., China) and PI for 15 min in the dark. Finally, apoptosis was quantified using a flow cytometer (FACSCalibur, BD Biosciences, San Jose, CA, USA). The data were collected from at least 10,000 cells derived from three independent experiments.

### Western Blotting Analysis

Total protein extracts were prepared from SH-SY5Y cells using radioimmunoprecipitation assay (RIPA) buffer. Equal amounts of protein samples (20–40 μg) were resolved on SDS-PAGE gels and transferred onto Immobilon polyvinyl difluoride membranes (GE Healthcare, Little Chalfont, UK) using standard protocols. The membranes were blocked with 4% bovine serum albumin (BSA) for 1 h at room temperature and probed with primary antibody against HIF-1α (1:2000; Cell Signaling Technology), BNIP3 (1:1500; Cell Signaling Technology), Beclin1 (1:1000; Abcam, Cambridge, MA, USA), LC3 (1:1000; Cell Signaling Technology), p62 (1:1000; Abcam), cleaved caspase-3 (1:2000; Cell Signaling Technology), GAPDH (1:2000; Cell Signaling Technology), or β-actin (1:1000; Beyotime Biotechnology, Haimen, China) at 4 °C overnight. After being washed with TBST (a mixture of tris-buffered saline and polysorbate 20), the blots were incubated with secondary goat anti-mouse or anti-rabbit antibody conjugated with horseradish peroxidase at room temperature for 2 h. After being washed three times, the proteins were visualized using an enhanced chemiluminescence kit (GE Healthcare). The proteins were quantified with ImageJ software and normalized to β-actin in parallel experiments.

### Knockdown of Beclin1 Expression in SH-SY5Y Cells

A short hairpin RNA (shRNA) construct was designed targeting a 21-nt sequence (5′-CCCGTGGAATGGAATGAGATT-3′) within the human Beclin1 gene (transcript variant: NM_003766.4). The shRNA hairpin sequence is shown in Table 1, in which TTCAAGAGA was used as the stem-loop junction and TTTTTT was used as the transcription terminator. GATCC was added to the 5′ end of the positive-sense primer to complement the cut of the *Bam*HI enzyme (sense sequence). A *Kpn*I site was added just behind the transcription terminator for identification of positive clones. CTTAA was added to the 5′ end of the negative-sense primer to complement the cut of the *Eco*RI enzyme (antisense sequence). After linearization with both *Bam*HI and *Eco*RI, Lenti-U6-GFP-puro vector was incubated at 16 °C overnight with T4 DNA ligase, the annealed double-stranded oligo, followed by transformation of *Escherichia coli* Stbl3 competent cells. Positive colonies were selected for *Kpn*I digestion and sequencing. A total 10 μg of Lenti-U6-shRNA-GFP-puro recombinant vector and 15 μL of lentiviral packaging mix (Catalog # LVP2MIX; iCARTAB Biomedical Co. Ltd., Suzhou, China) were subsequently mixed and transfected into HEK293T cells using LVTransm (Catalog # LVTran100; iCARTAB Biomedical Co. Ltd.). The supernatant containing recombinant lentivirus was harvested and concentrated at 20,000×*g* at 4 °C for 2 h, and the lentivirus was resuspended in PBS buffer and titrated by qPCR. A control lentivirus carrying scrambled shRNA was provided by iCARTAB Biomedical Co. Ltd.

**Table 1:** ShRNA sequence information

Sense sequence	gatccgCCCGTGGAATGGAATGAGATTTTCAAGAGAAATCTCATTCCATTCCACGGGTTTTTTCAATTGg
Antisense sequence	aattcCAATTGAAAAAACCCGTGGAATGGAATGAGATTTCTCTTGAAAATCTCATTCCATTCCACGGGcg

### Quantitative PCR

Total RNA was extracted from cells with TRIzol Reagent (Thermo Fisher Scientific) and transcribed into cDNA using a reverse transcription kit (Takara Biotechnology Co. Ltd., Dalian, China). The relative mRNA level of the target gene was detected using the Roche LightCycler® 480 Real-Time PCR system (Roche, Basel, Switzerland) according to the SYBR® Premix Ex Taq instructions (Takara Biotechnology Co. Ltd.). The primer sequences were as follows: forward 5′-CAGGAACTCACAGCTCCATT-3′ and reverse 5′-CATCAGATGCCTCCCCAATC-3′ for Beclin1 and forward 5′-ACCACACCTTCTACAATGA-3′ and reverse 5′-ATAGCACAGCCTGGATAG-3′ for β-actin. The results were quantified by the comparative CT (threshold cycle) method using β-actin as an internal control (Chen et al. 2017a).

### Statistical Analysis

Data were analyzed using SPSS 18.0 software (SPSS Inc., Chicago, IL, USA). All values are presented as the mean ± standard deviation. Statistical analyses for the comparison of two groups were performed using Student’s *t* test, and one-way analysis of variance followed by a post hoc least significant difference multiple comparison test was used for the comparison of more than two groups. *P* < 0.05 was considered statistically significant.

## Results

### HPC Protects SH-SY5Y Cells Against OGD/R Injury

**Fig. 1:**
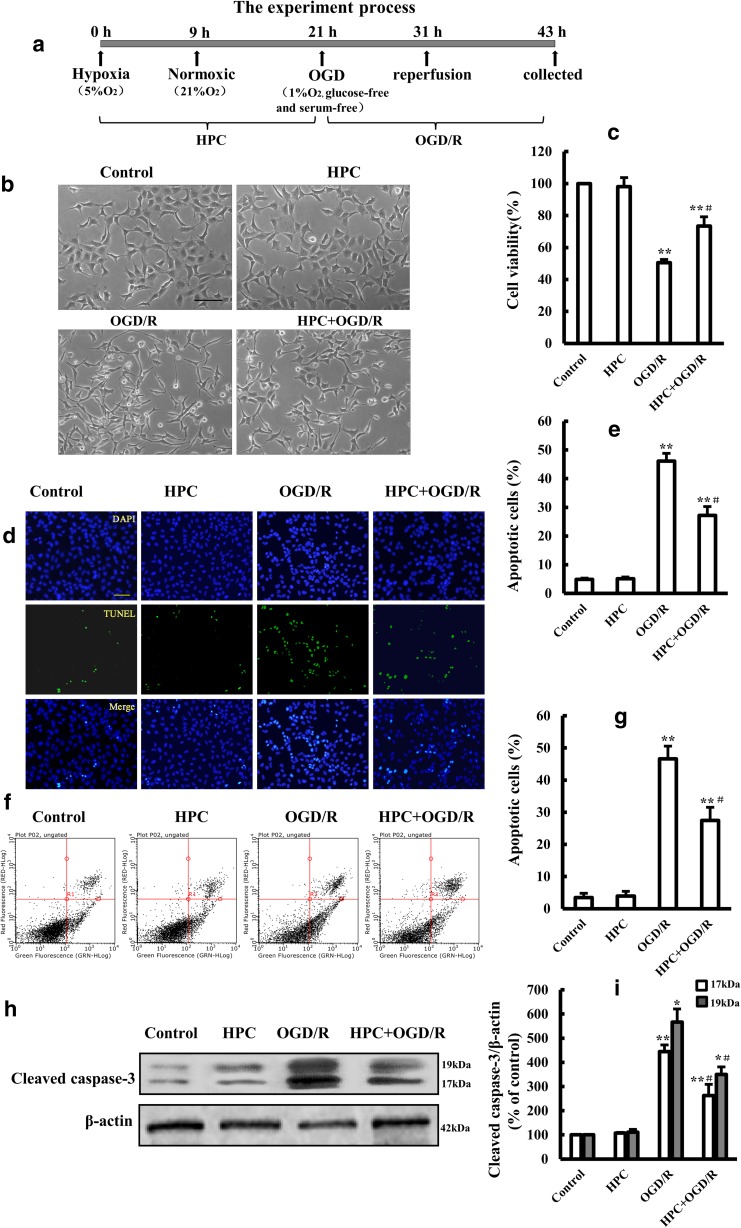
Effects of HPC on OGD/R-induced injury in SH-SY5Y cells. **a** Schematic diagram illustrating the HPC and OGD/R treatment of SH-SY5Y cells. **b** Representative microscopic images showing the cell morphology of control cells and cells subjected to OGD/R with or without prior treatment with HPC. Scale bar is 100 μm. **c** Summary of the mean cell viability determined by MTT from three independent experiments using six wells of cells in each experiment. **d**, **e** TUNEL analysis of cell apoptosis. Representative fluorescence microscopic images showing TUNEL staining (green) and DAPI nuclear staining (blue). Scale bar is 100 μm. **f**, **g** Analysis of cell apoptosis using flow cytometry under the conditions indicated above. **h**, **i** Representative Western blot showing the expression of cleaved caspase-3 proteins in SH-SY5Y cells under the indicated conditions and summary of the mean ± SD data from three independent experiments. *P < 0.05, **P < 0.01 vs. control group; ^#^*P* < 0.05 vs. OGD/R group

To investigate the effects of HPC on OGD/R-induced SH-SY5Y cells, we conducted experiments as illustrated in Fig. 1a. Cells were treated with hypoxia for 9 h followed by normoxia for 12 h, and thereafter, they were treated with OGD for 10 h followed by reperfusion for 12 h. Next, cell viability, apoptosis, and cleaved caspase-3 level were assessed. As shown in Fig. 1c, OGD/R treatment significantly decreased cell viability compared with that of control cells. The OGD/R-induced reduction in cell viability was significantly alleviated by HPC treatment (Fig. 1c). Additionally, OGD/R-induced cell apoptosis was significantly attenuated by HPC treatment (Fig. 1d–g). The cleaved caspase-3 level, which was robustly induced by OGD/R treatment, was significantly suppressed by HPC treatment (Fig. 1h, i). Our results demonstrate that HPC protected SH-SY5Y cells against OGD/R-induced injury.

### HPC Activates Autophagy in SH-SY5Y Cells

**Fig. 2:**
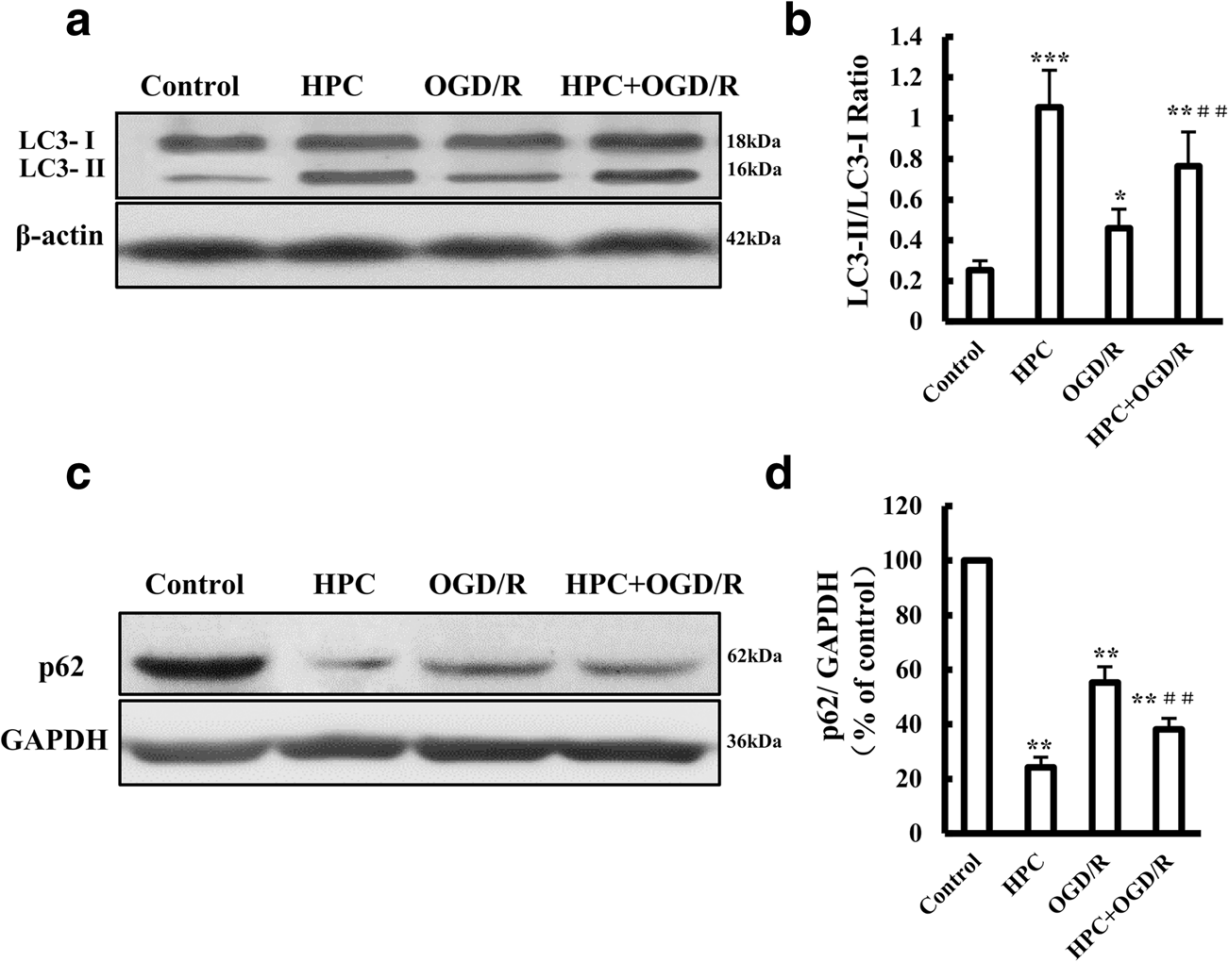
Effects of HPC on autophagy in OGD/R-treated SH-SY5Y cells. **a** Representative Western blot showing the expression of LC-3 protein in SH-SY5Y cells under the indicated conditions. **b** Summary of the mean data from three independent experiments. **c** Representative Western blot showing the expression of p62 protein in SH-SY5Y cells under the indicated conditions. **d** Summary of the mean ± SD from three independent experiments. *P < 0.05, **P < 0.01, and ***P < 0.001 vs. control group; ^##^*P* < 0.01 vs. OGD/R group

LC3-II is a hallmark protein of autophagy. Under normal conditions, LC3 protein exists in the cytosol as type I (LC3-I). When autophagy is activated, LC3-I is recruited to autophagosomes and subsequently converted to LC3-II (Mizushima 2004). As shown in Fig. 2a, b, the ratio of LC3-II to LC3-I was increased in cells treated with HPC or OGD/R compared with that in cells under normal conditions; the LC3-II/LC3-I ratio in cells subjected to HPC followed by OGD/R was also increased, exceeding the value for cells treated with OGD/R alone.

Protein p62, one of the selective substrates of autophagy, is localized at autophagosomes and constantly degraded by the autophagy-lysosomal pathway, which increases in response to a decrease in autophagic activity or a total loss of autophagic function (He et al. 2015). As shown in Fig. 2c, d, the p62 level was decreased in cells treated with HPC or OGD/R compared with that in cells kept under normal conditions. The p62 level in cells subjected to HPC followed by OGD/R was also decreased, reaching a value significantly lower than that in cells treated with OGD/R alone.

The results demonstrated that both HPC and OGD/R upregulated the autophagy level in SH-SY5Y cells. Moreover, HPC further increased the autophagy level, followed by OGD/R.

### Autophagy Is Involved in the Neuroprotection of HPC Against OGD/R-Induced Injury

**Fig. 3:**
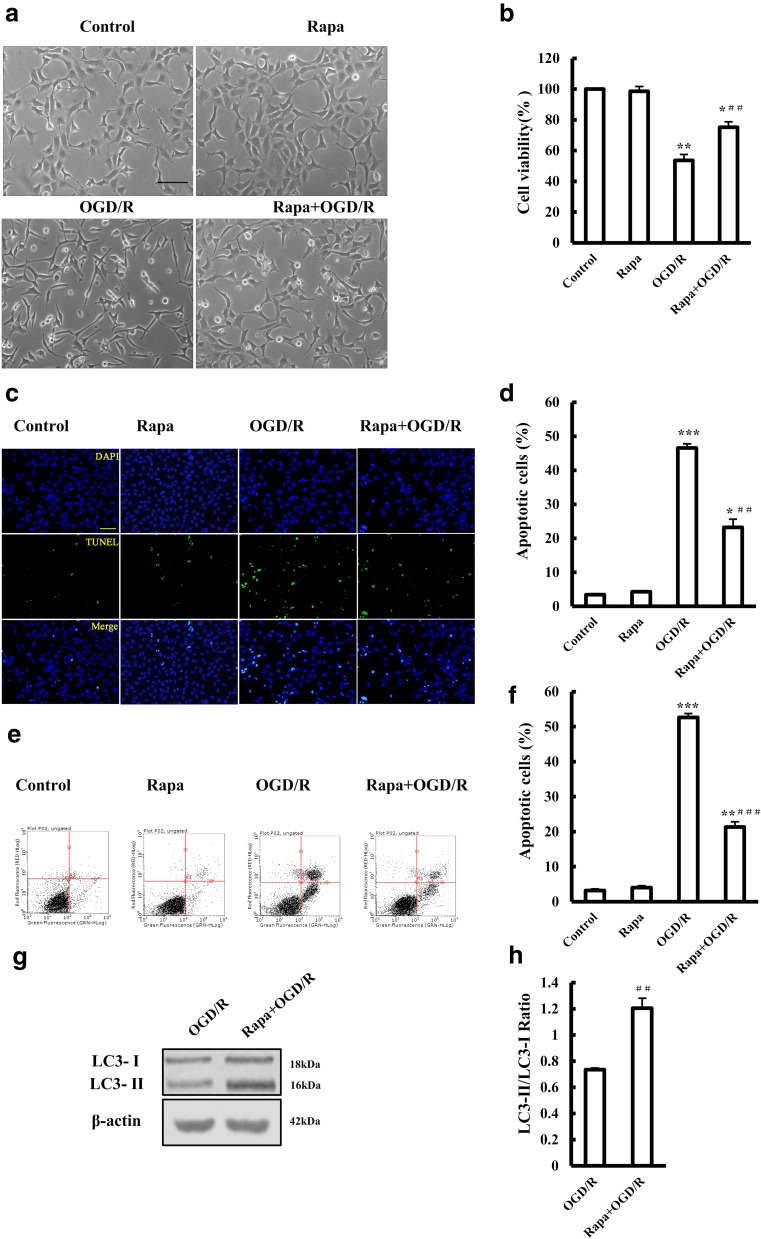
Effect of the autophagy agonist rapamycin on OGD/R-induced injury to SH-SY5Y cells. **a** Representative images showing the cell morphology of control cells, cells treated with 3 mM rapamycin (Rapa), and cells subjected to OGD/R with or without treatment using 3 mM Rapa 2 h before and during OGD/R. Scale bar is 100 μm. **b** Summary of the mean cell viability determined by MTT from three independent experiments using six wells of cells in each experiment. **c** Representative fluorescent images showing TUNEL staining (green), DAPI nuclear staining (blue), and merged images under the conditions shown in **a**. Scale bar is 100 μm. **d** Summary of the percentage of TUNEL-positive dead cells as shown in **c**, from three independent experiments with five different images analyzed in each experiment. **e**, **f** Analysis of cell death using flow cytometry under the conditions indicated above. **g**, **h** Western blotting showing the expression of LC-3 protein in SH-SY5Y cells. The mean results are from three independent experiments. *P < 0.05; **P < 0.01, and ***P < 0.001 vs. control group. ^##^*P* < 0.01 and ^###^*P* < 0.001 vs. OGD/R group

To examine whether autophagy was involved in neuroprotection by HPC against OGD/R-induced injury, we applied Rapa, an agonist of autophagy (Nair and Ren 2012). Treating the cells with Rapa increased OGD/R-cell viability (Fig. 3a, b). Additionally, OGD/R-induced apoptosis was significantly suppressed by Rapa (Fig. 3c–f). These results show that Rapa protects SH-SY5Y cells against OGD/R-induced injury through activating autophagy (Fig. 3g, h).

**Fig. 4:**
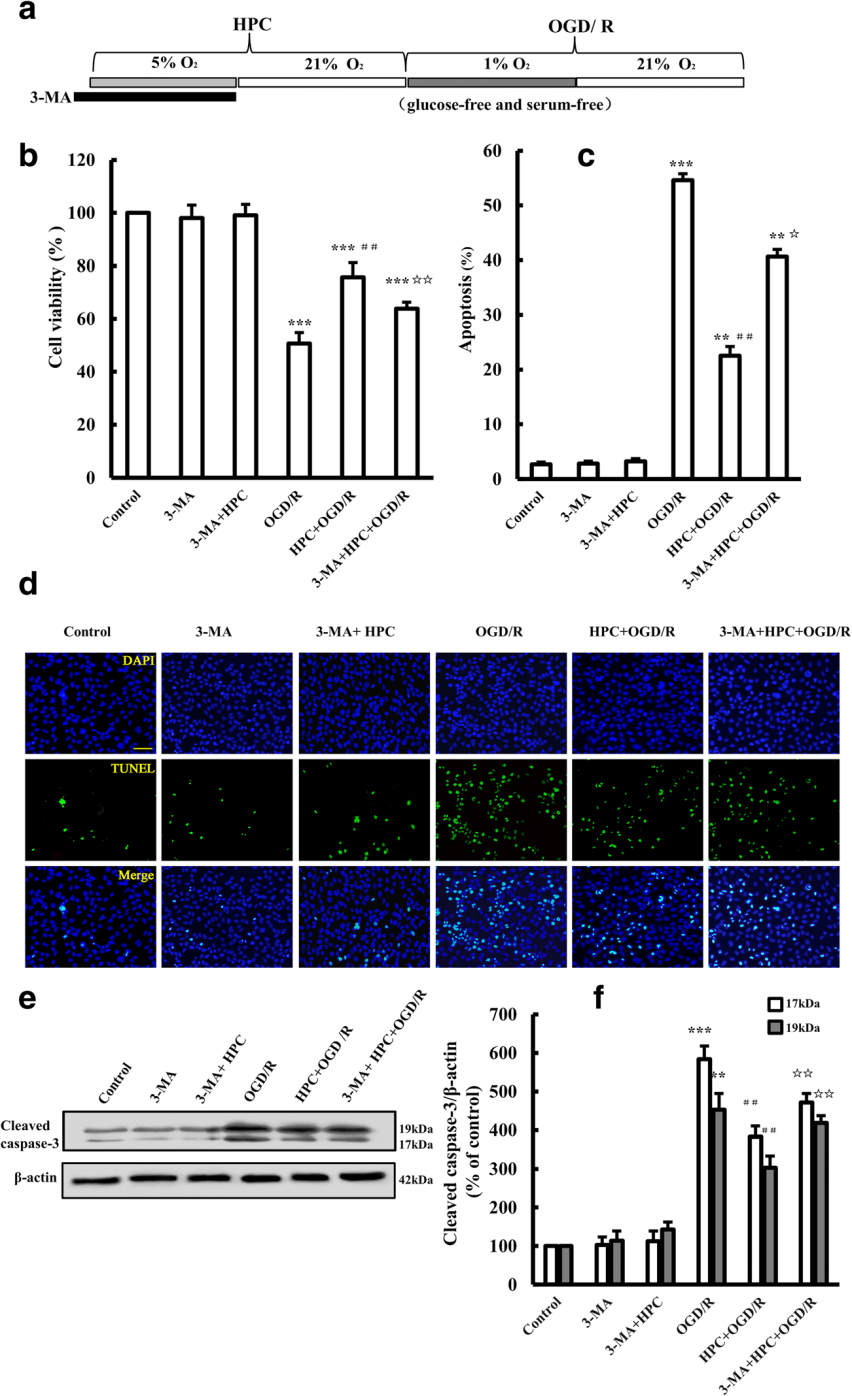
Effect of the autophagy inhibitor 3-MA on HPC-induced neuroprotection in SH-SY5Y cells. **a** Schematic diagram showing the treatment of SH-SY5Y with 10 mM 3-MA for 30 min before HPC followed by OGD/R. **b** Summary of the mean cell viability determined by MTT, under the conditions shown in **a**, from three independent experiments with six wells of cells in each experiment. **c**, **d** Representative fluorescent images showing TUNEL staining (green), DAPI nuclear staining (blue), and merged images under the conditions shown in **a**. Scale bar is 100 μm. Summary of the percentage of TUNEL-positive dead cells, as shown in **b**, from at least three independent experiments with at least three different images analyzed in each experiment. **e**, **f** Western blotting showing the expression of cleaved caspase-3 protein in SH-SY5Y cells. Data are shown as the mean ± SD from three independent experiments. **P < 0.01, ***P < 0.001 vs. control group; ^**##**^*P* < 0.01 vs. OGD/R group; ^☆^*P* < 0.05, ^☆☆^*P* < 0.01 vs. HPC + OGD/R group

The role of autophagy in HPC-induced neuroprotection was further studied by using 3-methyladenine (3-MA), an autophagy inhibitor. As shown in Fig. 4, administration of 5 mM (McFarland et al. 2013) 3-MA to cells treated with HPC + OGD/R significantly decreased cell viability and promoted cell apoptosis (Fig. 4b–d). Moreover, 3-MA significantly increased the cleaved caspase 3 level in cells treated with HPC + OGD/R (Fig. 4e, f). Our results indicate that inhibition of autophagy with 3-MA attenuated HPC-induced neuroprotection, providing evidence to suggest that autophagy is involved in the mechanism by which HPC protects SH-SY5Y cells against OGD/R-induced injury.

### HPC-Induced Autophagy Is Associated with the HIF-1α/BNIP3/Beclin1 Signaling Pathway in Control Cells

**Fig. 5:**
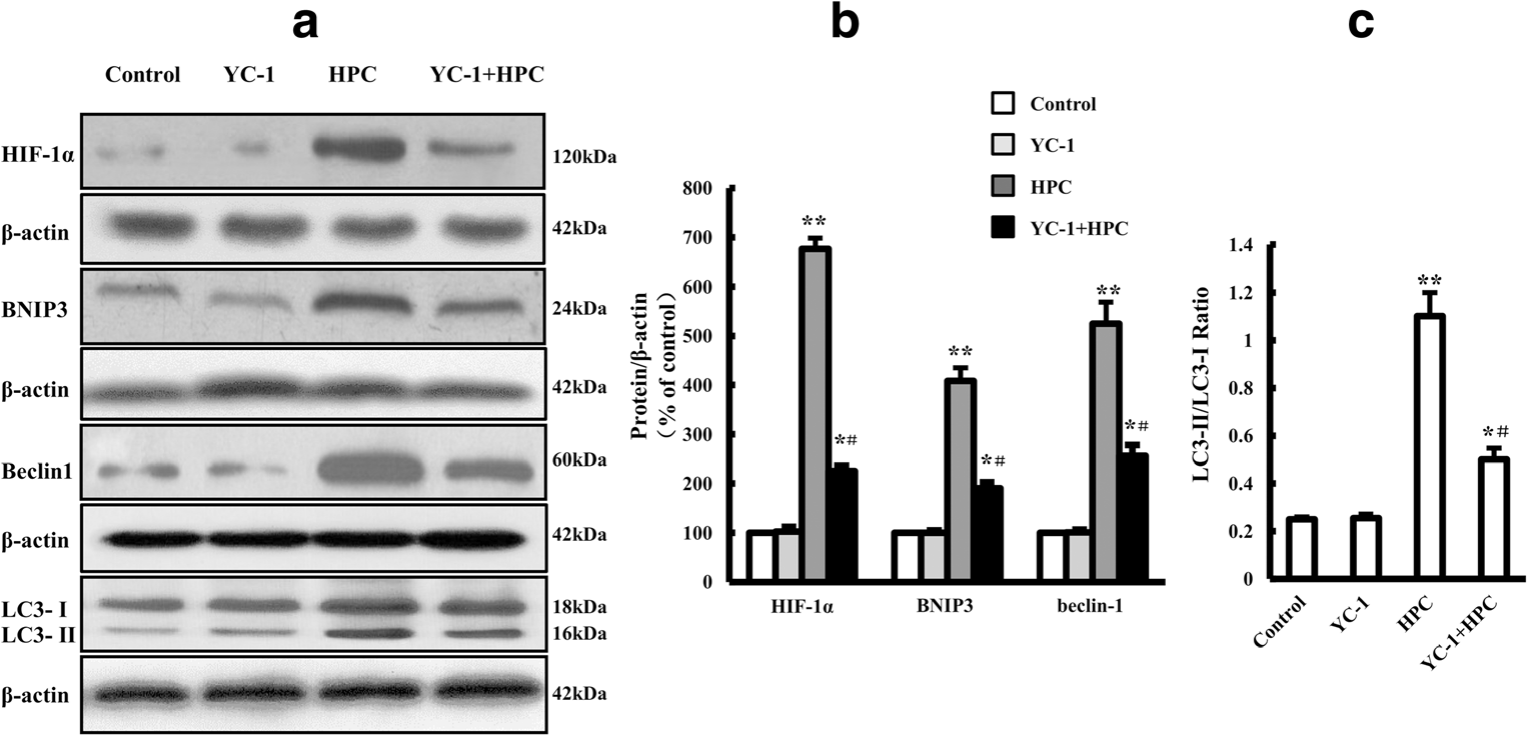
Effect of the HIF-1α inhibitor YC-1 on HPC-induced HIF-1α, BNIP3, Beclin1, and LC-3 protein expression in SH-SY5Y cells. **a** Western blots showing the expression of HIF-1α, BNIP3, Beclin1, and LC-3 proteins in SH-SY5Y cells. **b**, **c** Graph showing the mean results for relative optical density of protein bands on the blots, estimated using ImageJ software. Data shown as the mean ± SD from three independent experiments. *P < 0.05, **P < 0.01 vs. control group; ^**#**^*P* < 0.05 vs. HPC group

We next studied the effect of YC-1, an HIF-1α inhibitor (Rajoria et al. 2014), on the expression of HIF-1α/BNIP3/Beclin1 pathway components and LC-3 protein. As expected, the basal level of HIF-1α was relatively low, and treatment with YC-1 (10 μM) did not alter the levels of HIF-1α, BNIP3, or Beclin1 or the ratio of LC3-II to LC3-I. Compared with the control, the levels of HIF-1α, BNIP3, and Beclin1 (defined as 100%) and the ratio of LC3-II to LC3-I under HPC were 675.8 ± 22.5, 408.4 ± 26.7, 525.0 ± 43.3%, and 1.1 ± 0.1, respectively, demonstrating that HPC robustly increased the levels of HIF-1α, BNIP3, and Beclin1 and the ratio of LC3-II/LC3-I. Treating the cells with YC-1 reduced the expression of the above proteins (Fig. 5). These results suggest that HPC activated autophagy, likely through the activation of the HIF-1α/BNIP3/Beclin1 signaling pathway.

**Fig. 6:**
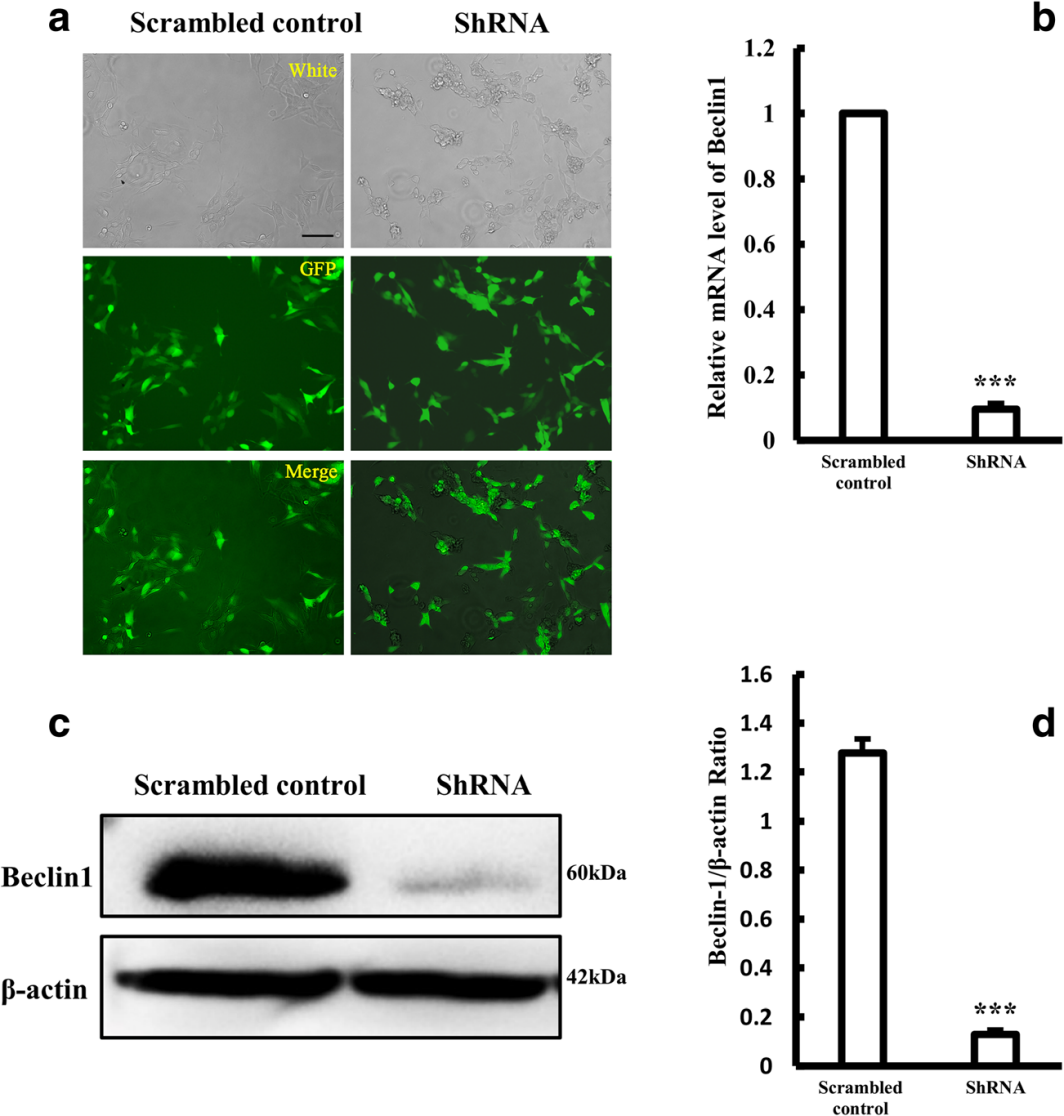
Construction of stable Beclin1 knockdown SH-SY5Y cell line. **a** GFP expression in SH-SY5Y cells following transfection with LV-Beclin1-shRNA. Scale bar is 100 μm. **b** Expression of Beclin1 mRNA in SH-SY5Y cells after Beclin1-shRNA transfection, measured by qPCR. Data are expressed as the relative value of the target gene compared with β-actin as the standard. **c**, **d** Western blotting showing the expression of Beclin1 protein in SH-SY5Y cells. Data are shown as the mean ± SD from three independent experiments. **P < 0.01; ***P < 0.001 vs. control group

**Fig. 7:**
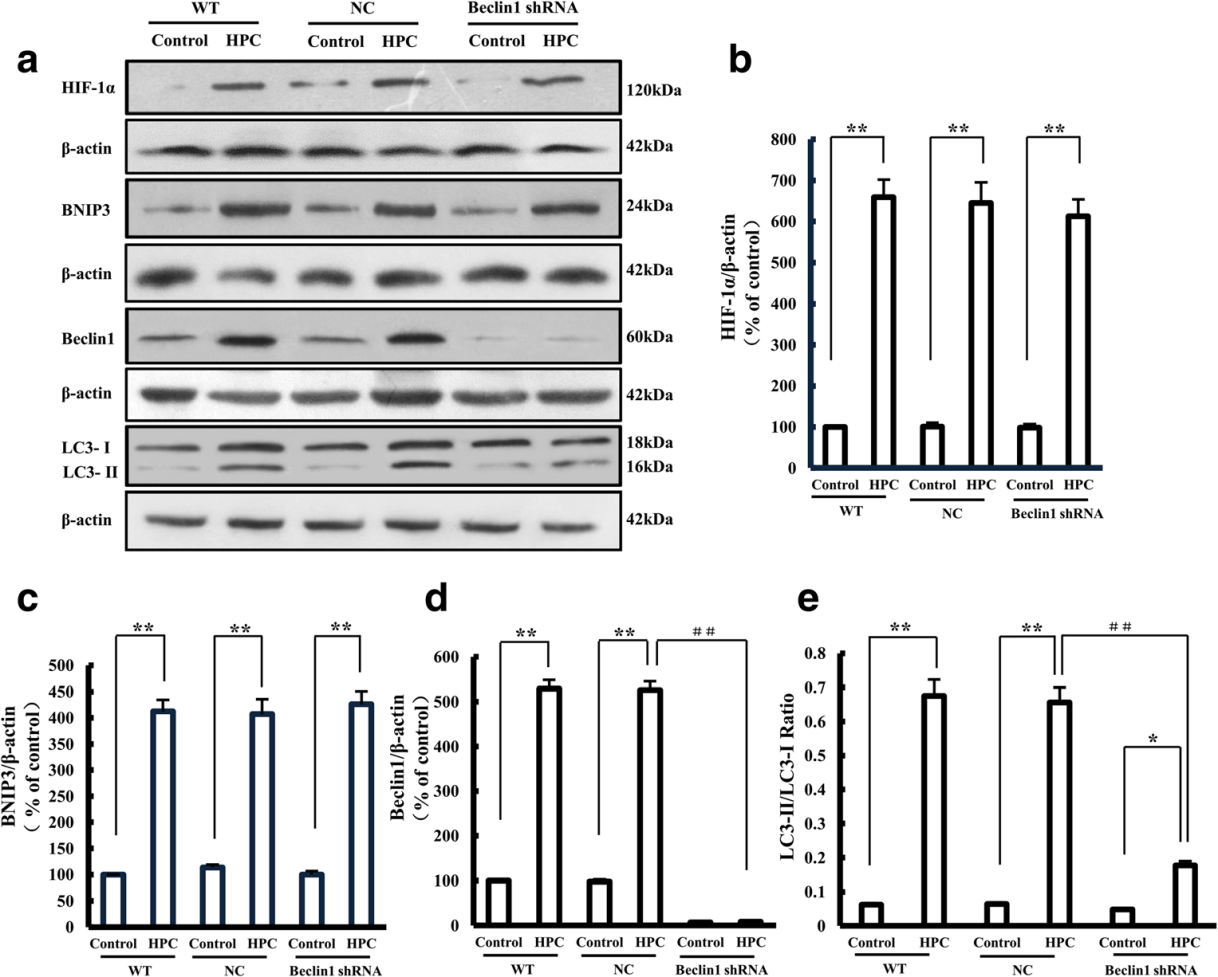
Effect of Beclin1-shRNA on HPC-induced HIF-1α, BNIP3, Beclin1, and LC-3 protein expression in SH-SY5Y cells. **a** Western blots showing the expression of HIF-1α, BNIP3, Beclin1, and LC-3 proteins after Beclin1-shRNA transfection of SH-SY5Y cells with HPC treatment. **b**–**e** Graphs showing the mean results for relative optical density of protein bands on the blots, estimated using ImageJ software. Data are shown as the mean ± SD from three independent experiments. *P < 0.05, **P < 0.01 vs. control group; ^**##**^*P* < 0.01 vs. HPC group of negative control (NC) cells

To further confirm the role of the HIF-1α/Beclin1 signaling pathway in HPC-induced autophagy, we stably knocked down Beclin1 by using a Beclin1-shRNA lentiviral vector (Fig. 6). The expression levels of HIF-1α and BNIP3 remained unchanged in Beclin1-knockdown SH-SY5Y cells after treatment with HPC, but the LC3-II/LC3-I ratio (0.18 ± 0.01) was significantly lower than that of negative control cells (0.66 ± 0.04), indicating that suppression of Beclin1 expression counteracts HPC-induced autophagy (Fig. 7). These results together with the results described above (Fig. 5) support the notion that HPC triggers autophagy through activating the HIF-1α/Beclin1 signaling pathway.

### HPC Enhanced Autophagy and the HIF-1α/BNIP3/Beclin1 Signaling Pathway in OGD/R-Injured Cells

**Fig. 8:**
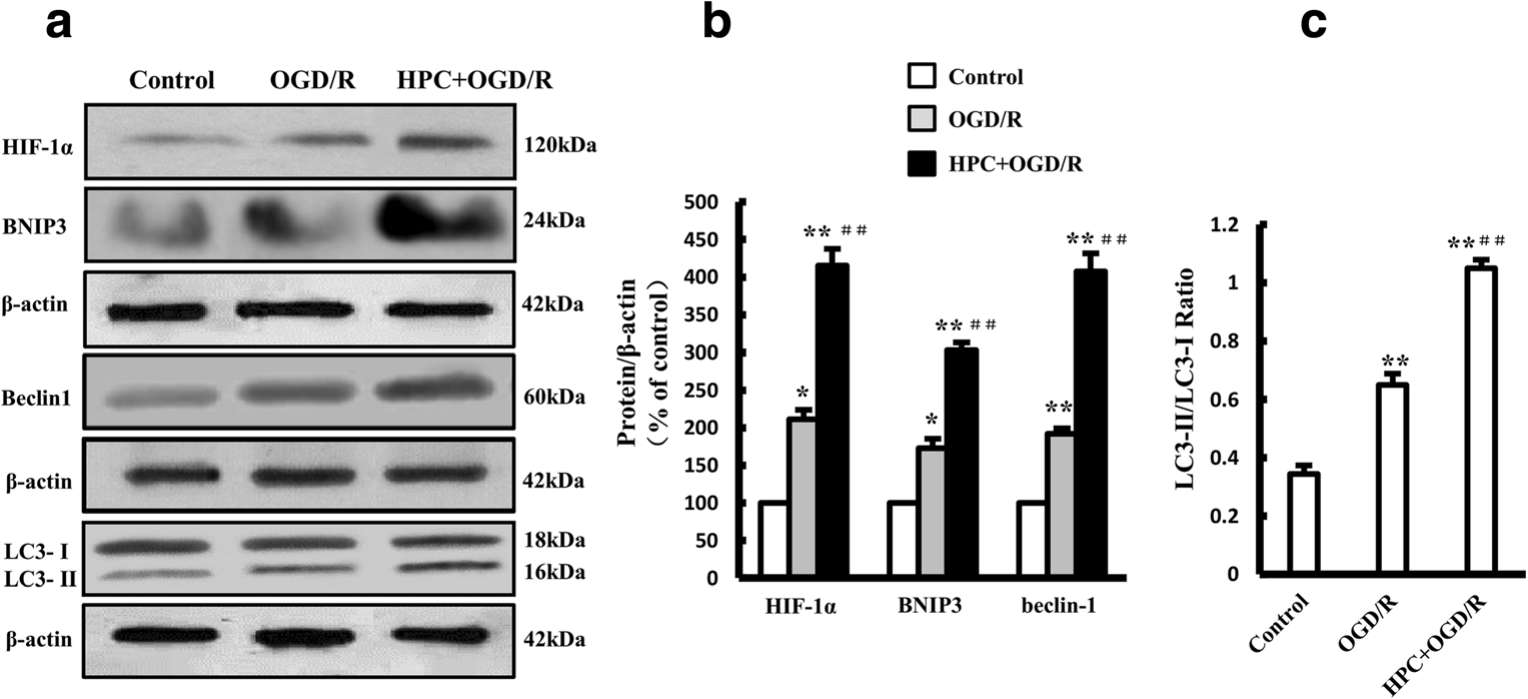
Effect of HPC on LC-3 and the HIF-1α/BNIP3/Beclin1 signaling pathway in OGD/R-treated SH-SY5Y cells. **a** Western blots showing the expression of HIF-1α, BNIP3, Beclin1, and LC-3 proteins. **b**, **c** Graphs showing the mean results for relative optical density of protein bands on the blots, estimated using ImageJ software. Data are shown as the mean ± SD from three independent experiments. *P < 0.05, **P < 0.01 vs. control group; ^##^*P* < 0.01 vs. OGD/R group

As shown in Fig. 8, the expression levels of HIF-1α, BNIP3, and Beclin1 were increased in cells treated with OGD/R (HIF-1α: 211.1 ± 12.9%; BNIP3: 172.9 ± 12.7%; Beclin1: 191.8 ± 7.3%) or HPC + OGD/R (HIF-1α: 415.5 ± 22.3%; BNIP3: 302.8 ± 10.3%; Beclin1: 407.5 ± 24.3%) compared with the levels in cells under control culture conditions. The ratio of LC3-II to LC3-I in cells subjected to OGD/R (0.65 ± 0.04) or HPC + OGD/R (1.05 ± 0.03) was also increased. Moreover, cells treated with HPC + OGD/R had higher expression levels of the proteins mentioned above than did cells in the OGD/R group, suggesting that HPC enhanced OGD/R-induced autophagy and HIF-1α/BNIP3/Beclin1 pathway activity.

### HIF-1α/Beclin1 Signaling Is Involved in the Neuroprotective Effect of HPC Against OGD/R-Induced Injury

**Fig. 9:**
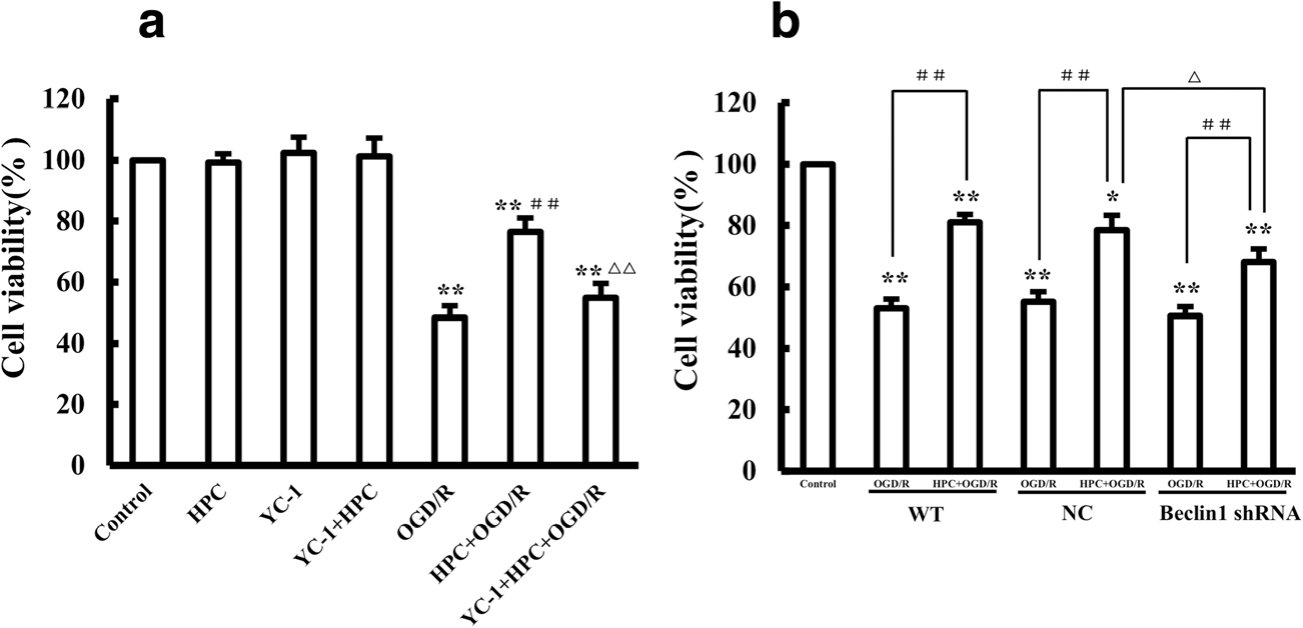
The HIF-1a/Beclin1 signaling pathway is involved in regulating the protective effect of HPC against OGD/R-induced injury. **a** Summary of the mean cell viability determined by MTT under the indicated conditions. **b** Effect of Beclin1 knockdown on the protective effect of HPC. Summary of the mean cell viability determined by MTT, from three independent experiments using six wells of cells in each experiment. Data are shown as the mean ± SD. *P < 0.05, and **P < 0.01 vs. control group; ^**#**^*P* < 0.05, and ^**##**^*P* < 0.01 vs. OGD/R group; ^△^*P* < 0.05, and ^△△^*P* < 0.01 vs. HPC + OGD/R group of negative control (NC) cells

The cell viability rates for the HPC + OGD/R and YC-1 + HPC + OGD/R groups were 76.4 ± 4.8 and 54.8 ± 4.7% (Fig. 9a). Treatment with YC-1 reduced cell viability in HPC + OGD/R cells, implying that the HIF-1α inhibitor counteracted the protective effects of HPC. Moreover, the cell viability of Beclin1-knockdown cells was 68.0 ± 4.3% in the HPC + OGD/R group, which was significantly lower than the viability of the negative control cells (78.5 ± 4.8%) (Fig. 9b); this finding implies that, compared with control cells, Beclin1-knockdown SH-SY5Y cells had reduced sensitivity to HPC, suggesting that Beclin1 knockdown attenuated the protective effects of HPC.

## Discussion

Ischemia reperfusion causes neuronal injury (Lin et al. 2016). SH-SY5Y human neuroblastoma cells are derived from the neural crest during neurodevelopment, exhibiting similar biochemical and functional properties to neurons (Shipley et al. 2016). In this study, a model of OGD/R-induced SH-SY5Y cell injury was established by using a hypoxic incubator, as in previous reports (Darshit and Ramanathan 2016; Guo et al. 2014; Zhu et al. 2016).

HPC refers to a nonfatal hypoxia exposure that confers a high degree of tolerance to subsequent severe hypoxia or other normally fatal stress, thereby offering a protective mechanism (Nikiforou et al. 2015). HPC has been confirmed to play a protective role not only in cardiovascular and renal systems but also in the central nervous system. However, the underlying mechanisms remain poorly understood. Recent study found that autophagy contributes to the neuroprotection of HPC against global cerebral ischemia and ethanol neurotoxicity in rats (Wang et al. 2013b; Zhan et al. 2017). Our study showed that HPC protects SH-SY5Y cells against OGD/R injury. A previous study reported that HPC did not cause neurotoxicity but induced autophagy (Shao and Lu 2012), and our data showed that HPC not only activates autophagy in normal SH-SY5Y cells but also further enhances autophagy in OGD/R-treated SH-SY5Y cells.

Autophagy, a mechanism of defense and stress regulation, plays an important role in maintaining cellular homeostasis and cell survival (Galluzzi et al. 2014). Autophagy has both pro-survival and pro-death functions, and recent studies have confirmed that enhancing autophagy has a protective effect against brain damage (Chen et al. 2012; Dai et al. 2017; Shen et al. 2016; Xie et al. 2016). Our results demonstrated that the autophagy agonist Rapa provided protection against OGD/R-induced injury. Moreover, 3-MA attenuated HPC-induced protection. Taken together, these results suggest that autophagy is critically involved in the protective effect of HPC, consistent with previous studies (Cui et al. 2015; Tzeng et al. 2010).

The HIF-1α signaling pathway can contribute to hypoxia-induced autophagy (Wu et al. 2015). HIF-1α increases the expression of BNIP3 under hypoxic conditions (Bellot et al. 2009; Li et al. 2013). In addition, BNIP3 is closely related to autophagy (Fu et al. 2018; Wang et al. 2013a); the activation of BNIP3 leads to the accumulation of LC3-II under hypoxia (Hanna et al. 2012). Beclin1 is an essential gene related to autophagy, and Beclin1-PI3KC3 complexes are key signaling complexes required for autophagosome formation (Wirth et al. 2013). Furthermore, mimicking hypoxic conditions with CoCl_2_ induces autophagy via activation of the HIF-1α/BNIP3/Beclin1 signaling pathway (Chen et al. 2017b).

Our results also showed that HPC activated autophagy while upregulating the HIF-1α/BNIP3/Beclin1 signaling pathway in SH-SY5Y cells, an effect that was counteracted by YC-1 treatment. Moreover, knockdown of Beclin1 expression suppressed HPC-induced autophagy without any effect on the expression of HIF-1α or BNIP3. Based on these findings and on previous studies (Bellot et al. 2009; Zhao et al. 2012), we conclude that HPC activates autophagy through the HIF-1α/BNIP3/Beclin1 signaling pathway in SH-SY5Y cells. In addition, HPC further activates autophagy and enhances HIF-1α/BNIP3/Beclin1 pathway in OGD/R-treated cells. Either treatment with YC-1 or knockdown of Beclin1 expression attenuates HPC-induced neuroprotection, suggesting that the HIF-1α/Beclin1 signaling pathway is involved in HPC-induced autophagy and neuroprotection.

In conclusion, our study reveals that HPC attenuates OGD/R-induced injury in SH-SY5Y cells by activating autophagy, and the HIF-1α/Beclin1 signaling pathway is involved in HPC-activated autophagy. Furthermore, HIF-1α and Beclin1 are also involved in HPC-induced neuroprotection.
